# Towards more sustainable oceans: A review of the pressing challenges posed by marine plastic litter

**DOI:** 10.1177/0734242X251313927

**Published:** 2025-02-13

**Authors:** Walter Leal Filho, Jelena Barbir, Julia May, Marta May, Julia Swart, Peter Yang, Maria Alzira Pimenta Dinis, Yusuf A Aina, Sara Bettencourt, Patricia Charvet, Hossein Azadi

**Affiliations:** 1 International Climate Change Information and Research Programme, Research and Transfer Centre ‘Sustainable Development and Climate Change Management’, Hamburg University of Applied Sciences, Germany; 2 Department of Natural Sciences, Manchester Metropolitan University, Manchester, UK; 3 Faculty of Life Sciences, Hamburg University of Applied Sciences, Hamburg, Germany; 4 Utrecht School of Economics, Utrecht University, Utrecht, The Netherlands; 5 Great Lake Energy Institute, Case Western Reserve University, Cleveland, OH, USA; 6 UFP Energy, Research, Innovation and Development Unit (FP-I3ID), University Fernando Pessoa (UFP), Porto, Portugal; 7 Marine and Environmental Sciences Centre (MARE), University of Coimbra, Edifício do Patronato, Coimbra, Portugal; 8 Department of Geomatics Engineering Technology, Yanbu Industrial College, Yanbu, Saudi Arabia; 9 Center for Global Studies (CEG), Department of Science and Technology, Portuguese Distance Learning University (UAb), Lisbon, Portugal; 10 Center for Environmental and Sustainability Research (CENSE), School of Science and Technology, NOVA University of Lisbon, Lisbon, Portugal; 11 MARE, Aquatic Research Network (ARNET), Regional Agency for the Development of Research, Technology and Innovation (ARDITI), Madeira, Portugal; 12 Faculty of Life Sciences, University of Madeira, Madeira, Portugal; 13 Programa de Pós-Graduação em Sistemática, Uso e Conservação da Biodiversidade (PPGSis), Departamento de Biologia, Universidade Federal do Ceará (UFC), Fortaleza, CE, Brazil; 14 Programa de Pós-Graduação em Engenharia Ambiental (PPGEA), Departamento de Engenharia Ambiental, Universidade Federal do Paraná (UFPR), Curitiba, PR, Brazil; 15 Department of Economics and Rural Development, Gembloux Agro-Bio Tech, University of Liège, Gembloux, Belgium

**Keywords:** Marine litter, bibliometric analysis, environmental policies, sustainability, governance

## Abstract

Marine littering is a global challenge and a significant threat to a sustainable planet, requiring comprehensive and effective mechanisms to address it in a comprehensive manner. This study reports on a bibliometric analysis that has identified the extent to which the topic has been explored in the international literature, by focusing on geographical scope, the emphasis on (micro)plastic litter and on policy measures. Additionally, as a complement to the assessment of the recent literature on marine plastic litter, this study reviews some case studies, identifying some trends on how to cope with this problem. The findings underscore the imperative for heightened research efforts in the context of marine littering. The literature reveals that unsustainable practices, the absence of robust policies and inadequate enforcement substantially contribute to the prevalence of marine plastic litter. Consequently, urgent action is essential, demanding the implementation of effective policies and frameworks. Encouraging nations to transition towards marine sustainability, particularly in terms of prevention and environmental awareness, is of paramount importance. To pave the way for a cleaner ocean for future generations, this study not only highlights the root causes but also offers suggested solutions. These solutions serve as valuable insights for researchers, innovators and policymakers worldwide, charting a course towards a more sustainable and litter-free marine environment.

## Introduction

Since the 1980s, an almost exponential increase in marine debris or litter has been reported (Ryan and Moloney, 1993). Two decades later, marine litter had been identified as one of the worst pollution problems faced by the world’s oceans (Law, 2017). About 26–91 million tonnes of litter entered the ocean between 1990 and 2015, with plastics accounting for the majority (61–87%) of the waste (Laufkoetter et al., 2020; Pham et al., 2014). Despite the fact that towards the end of the 1980s, a comprehensive understanding of the majority of marine litter effects had been achieved, prompting a shift in focus towards the exploration of viable solutions it took more than 30 years for marine litter to be recognized as a critical global issue for a healthy and sustainable planet and incorporated into policy instruments (Laufkoetter et al., 2020). Additionally, more researchers and experts are highlighting its effects on the biota, socio-economics and health of humans (Jorgensen et al., 2017).

Marine litter, defined as any solid substance, whether intentionally created, generated or processed, encompassing all items that have been discarded, disposed of or neglected within the marine and coastal environment is an important environmental hazard across the world (De-la-Torre et al., 2023; UNEP, 2021b). Moreover, natural weather events such as heavy rains and storms can lead to the movement of a multitude of anthropogenic (plastic) waste from coastal areas into the aquatic environment (Gündoğdu et al., 2018; Nakajima et al., 2022). Marine litter, particularly plastic litter, negatively impacts marine organisms through various ways such as ingestion, toxic contamination and entanglement (Sciutteri et al., 2023). Thus, the contamination caused by marine plastic litter is an urgent threat to marine biota (Claro et al., 2019; Ugwu et al., 2021), as well as for human health due to plastic’s accumulation in the food chain (Waring et al., 2018; Yuan et al., 2022).

Marine (plastic) litter can be classified as land-generated and ocean-generated litter based on size (nano, micro, meso and macro) and according to the origin of the items (Diem et al., 2023; Haseler et al., 2018; Wayman and Niemann, 2021). Generally, sea-based origin refers to debris released directly into the ocean by marine activities, while land-based origin includes activities that result in litter on land or a coast and ultimately ending up in the sea (Anfuso et al., 2015). Plastics are a significant component of marine litter, and large amounts of plastic are increasingly concentrated in marine ecosystems and environments (Jambeck et al., 2015; Napper and Thompson, 2020), particularly microplastics (<5 mm) which are more likely to be ingested by various marine biota (Porcino et al., 2022; Prinz and Korez, 2020). According to IUCN (2021), plastic accounts for 80% of all marine litter. Moreover, the omnipresence of marine litter in the environment is demonstrated by multiple incidences of floating litter from marine litter patches (Biermann et al., 2020; Galgani et al., 2015). Indeed, marine litter has been identified to be visible on coasts (Nelms et al., 2019), on shorelines of distant islands (Vogt-Vincent et al., 2023), in sea surface waters (Lacerda et al., 2019; Russell and Webster, 2021) and to be predominant in the deep sea (Barrett et al., 2020; Woodall et al., 2014), in arctic sea ice (Kanhai et al., 2020; Obbard et al., 2014), at sea around Antarctica (Barnes et al., 2010; Lacerda et al., 2019) and in polar waters in the Arctic (Bergmann et al., 2022).

Plastic entered into the marine ecosystem can be widely distributed due to its durability and buoyancy (Napper and Thompson, 2020). Microplastic can be found at sea depths (Harris, 2020; Harris et al., 2023; Vega-Moreno et al., 2021) and in sediments (Harris et al., 2023; van Emmerik et al., 2022). Furthermore, the penetration of plastic into marine habitats has numerous negative economic and environmental implications (Cerrillo-Escoriza et al., 2023; Díaz-Mendoza et al., 2020; Watt et al., 2021), restricting recreational uses and resulting in a decline in touristic value. Moreover, fish farms and other forms of aquaculture are among the numerous economic activities that experience losses (UNEP, 2014, 2021b; Wu et al., 2023). UNEP (2021b), for example, indicates that in the Mediterranean Sea annually US$696 million are lost in three major sectors: fisheries and aquaculture, shipping and tourism.

From an environmental perspective, marine litter impacts the marine environment and marine life (Gall and Thompson, 2015; Omeyer et al., 2023; Rochman et al., 2016). In this regard, the entanglement caused by plastic and ingestion of marine litter by fauna is highly critical (Collard and Ask, 2021; Costa et al., 2022; Rochman et al., 2015). Seabird species and various species of fish ingest marine litter due to misidentification of litter items as natural prey (CBD, 2012; Gregory, 2009; Prinz and Korez, 2020). Consequently, exposure to marine litter has an influence on species’ health, reproductive capabilities and mortality rate (Gall and Thompson, 2015; Thushari and Senevirathna, 2020).

Indeed, the accelerating use of single-use plastics (SUP) (Schnurr et al., 2018), unrestricted contamination with litter, accompanied by inadequate waste management and recycling programmes are considered the central causes for the concentration of marine litter in the oceans (IUCN, 2021). In various coastal countries, improper management of solid waste leads to 1.7–4.6% of the total plastic waste produced entering the sea (Jambeck et al., 2015), which increases marine litter. In the lack of effective regulations and enforcement measures, the present reliance on plastics and business-as-usual will contribute to raise these values. UNEP (2021b) forecasted that 48 million tonnes of plastic to reach the seas annually by 2030.

Despite initiatives to decrease marine litter contamination (e.g. European Commission, 2008, 2018, 2021; UNEP, 2016, 2021b), decisive and wide-ranging measures are still lacking. Therefore, actions to reduce marine litter must involve concerted efforts across nations, disciplines and stakeholder groups, considering a variety of pathways (GIZ, 2018; Secretariat of the Pacific Regional Environment Programme, 2014). The general public, commercial users of the ocean and beaches, waste management organizations, industry (e.g. designers of products and manufacturers), legislators, instructors, environmental non-governmental organizations and civil society organizations, national, regional and local government must all be involved in actions. Considering that marine litter is one of the main causes of contamination of the oceans, research and government surveys should be used to develop a better knowledge of the origins of marine litter so that focused management measures could be implemented. This study intends to provide a reference for the current marine litter situation, identifying common research lines in the literature, as well as current gaps which need to be addressed in order to facilitate a more effective implementation of policies to tackle the marine plastic litter problem. For this purpose, this research describes trends in marine litter literature. This study reported on a bibliometric analysis to identify the extent to which the topic has been explored in the international literature, and the attention that has been devoted to how to handle the marine littering problem. Additionally, this study relies on 10 case studies of island states. Gaps and challenges that were previously created and continuing engineering initiatives were recognized. This study also proposes a future priority for the management of marine litter, which is currently contaminating the seas and oceans, in order to attain and preserve a healthy ocean and the globe.

## Methods

This article reviews the academic knowledge on marine litter in terms of geographical scope, the emphasis on (micro)plastic litter and on policy measures. For this purpose, a statistical literature analysis and a presentation of case studies were conducted. For the statistical analysis, a dual approach was carried out, comprising a bibliometric analysis and a bibliometric systemic review of the main focus of the literature research on marine litter. The bibliometric analysis of marine litter research includes two steps, (i) a theme-based visualized bibliometric analysis of the research on marine litter and (ii) a manual bibliometric analysis of the related studies published. The visualized bibliometric analysis was applied to the related studies published in the period 1975–2022. For this analysis, a broad-based search string (‘marine litter’ OR ‘marine plastics’ OR ‘marine contaminants’ OR ‘marine debris’) AND (‘marine organisms’ OR ‘marine environment’ OR ‘marine ecosystems’) was used to collect data from the ScienceDirect database, one of the most popular bibliographic databases in the world. To gain a comprehensive understanding of the structure and thematic focus of research related to the environmental impacts of marine litter on the global oceans, the text mining and visualized bibliometric analysis abilities of VOSviewer (version 1.6.16), a widely used research tool for bibliometric analysis (van Eck and Waltman, 2020), was used. It allows to explore key research focus areas and create, visualize and explore maps of bibliometric network data by analysing bibliometric essentials of research publications indexed in specific databases, among different outputs.

The second step was a manual bibliometric analysis of marine litter research. In this step, the string ‘marine litter’ in the article title, abstract and/or keyword was used to collect data on the marine litter journal publications in the period 2018–2022. This study considered only ‘Review articles’ and ‘Research articles’ as search criteria. To be consistent, this study again used the ScienceDirect database to retrieve the list of articles. Based on the collected list of articles, this study explored co-word analysis, affiliation country of main authors, region of study and links with the citation metric and *h*-index in more detail for these years. Citation metric and *h*-index were retrieved from Google Scholar. Data collection was done in end-April 2023.

The analysis of marine litter policies was conducted through an expert-driven review of the most recent journal publications on marine litter development of marine litter conventions, laws and policies published from 2018 to 2022.

In addition to the statistical analysis, case studies were analysed to provide a comprehensive understanding of the main challenges in marine litter, and as such to use them to formulate recommendations of areas to address in future marine litter studies. A total of 10 case studies were gathered from the literature to illustrate the problems and challenges faced by island states. Because of their size, island states often have limited capacity to correctly dispose of waste, resulting in waste ending-up in the ocean. Therefore, island states provide a good basis to explore possible solutions to marine litter.

## Results and discussion

### Bibliometric analysis

#### Visualized bibliometric analysis of marine litter research, 1975–2022

The search for the visualized bibliometric analysis generated 7340 journal publications on marine litter published from 1975 to 2022, which revealed that the marine litter research grew by 118% annually over the last 26 years from 1997 to 2022. This represents an annual increase of 129.7% over the last 10 years (2012–2022), or an annual increase of 132.4% in the last 5 years (2017–2022).

**Figure 1.**
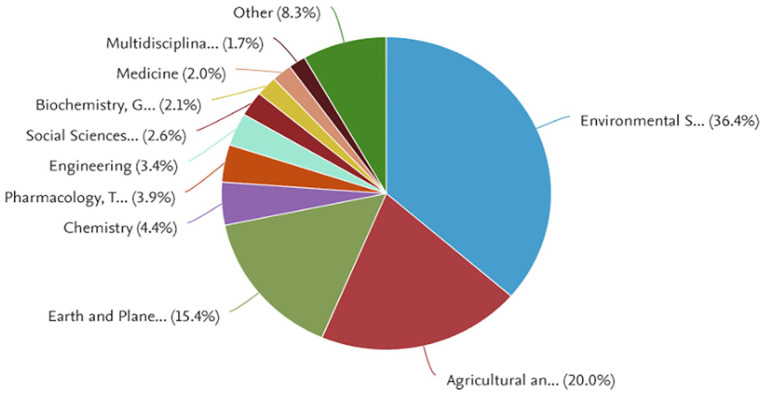
Research area distribution on marine litter from 1975 to 2022 (*n* = 7340 journal publications).

The statistical analysis shows that an overwhelming majority of journal publications on marine litter (72%) were focused on Environmental Science (36.4%), then Agricultural and Biological Sciences (20.0%) and lastly Earth and Planetary Sciences (15.4%) (see Figure 1).

**Figure 2.**
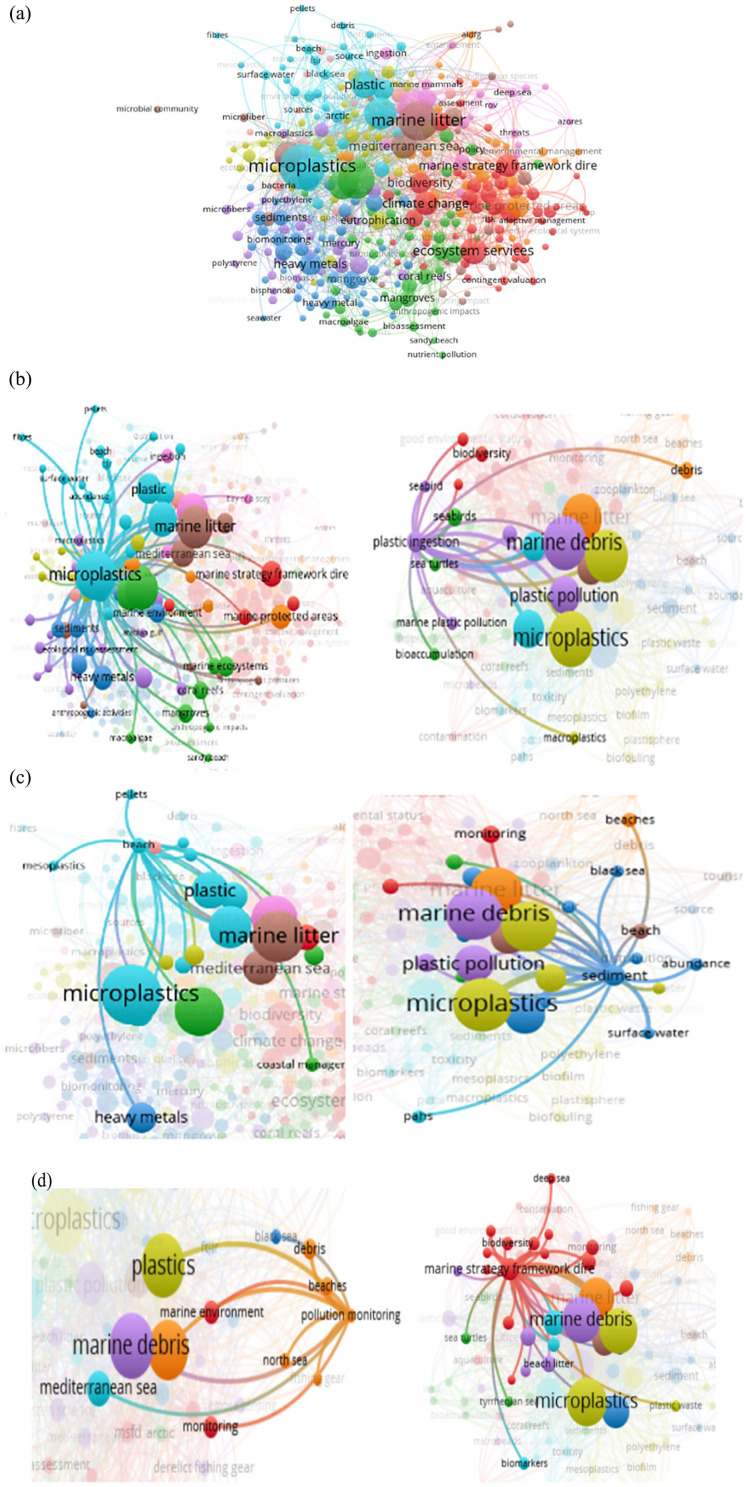
(a) Bibliometric networks of marine litter research (1975–2022). (b) Bibliometric networks of plastic and microplastic pollution; plastic ingestion (1975–2022).(c) Bibliometric analysis on beach marine litter and regional distribution (1975–2022). (d) Research map of marine pollution monitoring and strategy framework (1975–2022).

Figure 2 shows the visualized output of bibliometric analysis of the marine litter research, where the node size is proportional to the occurrence frequency and the link width is proportional to the strength of the connection. Terms that are closely related to each other form thematic clusters. Although Figure 2(a) shows a full map of interconnected marine litter research topics, Figure 2(b)–(d) shows connections of related sub-research topics on marine litter.

#### Manual bibliometric analysis

The manual bibliometric approach led to the selection of 619 articles published in 81 different peer-reviewed journals, being the journal *Marine Pollution Bulletin* the most popular journal, with around 45.7% of the journals in marine litter being published in this journal. The second most popular journal, *Science of the Total Environment*, covered 12% of the articles. In total, 46 out of 619 of the articles have been published in 46 separate journals, indicating a wide dispersion of the articles in terms of publication target. Table 1 provides an overview of the eight most popular journals in this respect.

**Table 1.** Distribution of articles in marine litter according to specific journals, 2018–2022 (*n* = 619).

Journal	Number	Percentage
*Marine Pollution Bulletin*	283	45.7
*Science of the Total Environment*	75	12.1
*Environmental Pollution*	51	8.2
*Ocean & Coastal Management*	16	2.6
*Waste Management*	9	1.5
*Ecological Indicators*	9	1.5
*Journal of Hazardous Materials*	8	1.3
*Estuarine Coastal and Shelf Science*	8	1.3

The analysis based on the main author’s affiliation country indicates a concentration of research on the topic which is produced in European countries. In particular, it is seen that Italy and Spain together account for close to 30% of all journal articles in the years 2018–2022 (Table 2). This also relates to the region of study of the published articles (Table 3), with Europe being the continent that is mostly studied, despite the majority of global studies (18.3%). This suggests that much of the current understanding about marine litter relates to developments in certain parts of the world, in particular, Europe.

**Table 2.** Distribution of articles in marine litter according to main author’s affiliation country, 2018–2022 (*n* = 619).

Main author’s affiliation country	Number	Percentage
Italy	90	14.5
Spain	73	11.8
UK	47	7.6
China	30	4.8
Portugal	29	4.7
Brazil	27	4.4
Germany	22	3.6
Greece	21	3.4
France	20	3.2
Australia	17	2.7

The table contains the 10 top nationalities. The total number in the table is 376, comprising about 61% of the journals. The remaining 39% of the journals comprise 60 other different nationalities.

**Table 3.** Distribution of publications in marine litter according to the region of study, 2018–2022 (*n* = 619).

Study region	Number	Percentage
Global	113	18.3
Mediterranean Sea/Area/Beach	69	11.1
Italy	23	3.7
Spain	23	3.7
Brazil	19	3.1
Portugal	17	2.7
China	16	2.6
Baltic Sea	12	1.9
Europe	12	1.9

The table contains the nine top regions of study. The total number in the table is 304, comprising about 49% of the journals. The remaining 51% of the journals comprise over 115 different regions.

**Figure 3.**
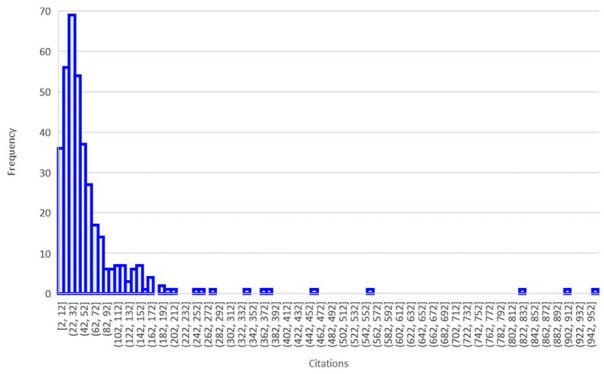
Frequency of articles on marine litter with respect to the citations’ number (2018–2020).

In terms of citation, the search focused on articles published in the years 2018–2020. The average number of citations (retrieved end of April 2023) was 61.3, although there is a significant dispersion. One article, which proposes a new definition for microplastics based on methods for describing and identifying microplastics (Frias and Nash, 2019), had received over 950 citations by the end of April 2023. The second most cited article, from Prata et al. (2020), had received more than 910 citations in the same period. Only 21 out of 619 articles had received more than 150 citations. A common feature of the most cited articles is that most of the main author’s affiliation relates to Europe and investigates either European regions or globally. This finding reinforces our previous one that the current understanding is centred in European research. Moreover, separating the articles from 2018 to 2020 in two main groups, that is, at least 50 citations, and 0–50 citations, shows that the most cited publications have a larger frequency of key terms related to policy, impact, regulation, organism, debris, plastic and microplastic of marine litter (Figure 3 and Table 4), suggesting an interest in the literature on findings related to these specific terms. Overall, the term ‘microplastic’ appears in more than 75% of the articles either in the abstract, keywords or in the highlights.

**Table 4.** Percentage of articles with the key terms in abstract, keywords or highlights.

Key term	2018–2020 articles with at least 50 citations by the end of April 2023 (%)	2018–2020 articles with less than 50 citations by the end of April 2023 (%)	All 2018–2022 articles (%)
Governance	1.525	1.67	2.42
Policy	14.39	7.92	13.25
Impact	31.06	29.58	29.89
Agreement	1.52	1.25	1.45
Regulation	3.79	1.67	2.10
Assessment	29.55	34.58	32.79
Monitoring	22.73	23.33	22.94
Organism	22.73	8.75	12.76
Ecosystem	18.94	20.00	20.84
Contaminant	6.06	5.00	4.85
Debris	41.67	35.00	35.86
Plastic	79.55	67.92	75.12
Microplastic	46.21	30.00	32.47

There were 132 articles in the period 2018–2020 which had received at least 50 citations by the end of April 2023. There were 240 articles in the period 2018–2020 which had received less than 50 citations by the end of April 2023.

Finally, the *h-index* of the first author (when available), which resulted in a total of 394 data points, was analysed. Interestingly, the correlation with the number of citations of the article is not high (0.05), suggesting that authorship is not a key determinant of readership. The highest observed *h*-index was 178, the second-highest 84 and the third highest was 70. This finding suggests that author’s academic performance is not a main determinant of readership.

### Case studies

Furthermore, we present 10 case studies of island states (see Table 5 for an overview). These case studies illustrate gaps and challenges faced in terms of marine litter.

**Table 5.** Data of 10 island States in the world.

Island nation	Population (2022)	GDP (2022) billion US dollar	Existence of policies to handle marine littering	Reference
Barbados	281,995	5.087	Reliable infrastructure and methods for the collection, sorting, processing and disposal of solid waste. Opportunities for increasing recycling efforts, educational opportunities to support a circular economy and continuous scientific study to support better decision-making	Clayton et al. (2021); Graham (2023)
Jamaica	2,827,377	17.10	Successful government action to decrease back on the use of plastic Bans for the most problematic plastics Educational programmes to increase awareness	Clayton (2021); Clayton et al. (2021)
Saint Martin	31,791	1.394	Improved disposal for garbage Public awareness-raising efforts to clean up the beaches Local government initiatives to reduce trash Increase local behavioural change in favour of the environment	Al Nahian et al. (2023); Bhuyan et al. (2019)
Nosy Be	81,115	13.964	Waste recycling and recovery efforts to promote resource sustainability and the reduction of environmental, economic and social repercussions	Diamant et al. (2021); Ferronato et al. (2023)
Cape Verde	593,149	2.31	Improving community participation, creating awareness campaigns and implementing ocean literacy programmes to prevent garbage from entering the ocean. Comprehensive ocean and coastal management strategies to decrease marine litter in the ocean and marine habitats. Appropriate management to ensure the protection and conservation of maritime biodiversity and ecosystems	Ferreira et al. (2021); Weidlich and Lenz (2022)
Mauritius	1,262,523	29	Thorough education and understanding of the issue of marine trash among tourists Smoking-related pollution on beaches should be avoided. Reducing pollution sources from recreational and beach activities through the establishment and enforcement of tighter littering laws	Mattan-Moorgawa et al. (2021); Seeruttun et al. (2023)
Fiji	929,766	4.296	Determine the most effective policy choices for dealing with plastic trash. Consider the involvement of stakeholders in reducing marine trash, such as non-governmental organizations, academics and communities.	Paris et al. (2022); Suratissa and Rathnayake (2017)
Samoa	222,382	832	Increase the collection of municipal solid garbage. Reduce plastic waste through increasing plastic collection and recycling. Integration of several waste management policies	Asari et al. (2019); Polidoro et al. (2017)
Cousine in Seychelles	490	14	Government action that is effective in addressing pollution issues Include the local community in frequent public awareness efforts and significant clean-ups of the beach.	Dunlop et al. (2020); Evans et al. (2022)
Cook	17,011	252	Infrastructure for the disposal of waste should be improved. Reduce waste and slow the increasing usage of single-use plastics Provide recommendations for waste reduction and ‘downstream’ waste management.	Dunlop et al. (2020); Farrelly et al. (2021)

#### The Windward Islands in the south of the Eastern Caribbean archipelago

The Windward Islands, which are the largest group of islands in the Eastern Caribbean, are formed up of Barbados, Dominica, Grenada, St. Lucia and St. Vincent and the Grenadines (SVG). Among other climatic risks, hurricanes and storm surges pose a threat to the coastlines and related activities each year (Ferdinand, 2013; Miranda et al., 2021). One of the factors is the geographical footprint of the Windward Islands, which is not only challenged by managing domestic solid waste, but is also impacted by marine and terrestrial debris from other places via ocean currents (de Scisciolo et al., 2016). According to Graham (2023), the internal waste production of the Windward Islands mostly takes the form of plastic trash produced by vacationers and leisure activities, as well as other inappropriate disposal behaviours including littering, illegal dumping and abandoned fishing gear. But the Windward Islands are affected by debris from the North Atlantic Gyre and its main currents, in addition to waste from the south equatorial currents over Venezuela that enter the North Atlantic gyre (Hurley et al., 2019; World_Bank, 2019).

#### Jamaica island

Jamaica is the third-largest of the Greater Antilles in the Caribbean Sea, with a population of about 2.73 million and a land area of over 10,900 km^2^. Jamaicans produce 800,000 tonnes of residential trash annually (World_Bank, 2021). About 15% of the trash that the National Solid Waste Management Authority (NSWMA) collects is made up of plastic (UNEP, 2021c). In the past, SUP bags were commonly used and sold in stores in Jamaica. However, their excessive use and subsequent careless disposal led to blocked sewage lines. This led to significant economic costs and a heavy burden on the NSWMA (CPRI, 2017). The bulk of Jamaica’s solid waste cannot be collected and disposed of by the NSWMA in an effective and sustainable manner. Solid waste management (SWM) coexists with other pressing economic and social issues including budget deficits, crime, poverty and unemployment (CPRI, 2017). Due to fragmentation and dispersion, part of Jamaica’s solid waste eventually finds its way into the water as only 75% of it gets disposed of in designated disposal sites. Additionally, these open dumps expose plastic and other solid trash to the elements, animals and uncontrolled waste disposal companies (UNEP, 2021c). The other 25% is either burned or left as garbage, most of which regularly makes its way into drains, rivers, gullies, beaches, and ultimately the ocean (Clayton, 2021).

#### Saint Martin Island

Saint Martin Island is located in the northeastern part of the Bay of Bengal. Saint Martin Island, a sedimentary island in Bangladesh, has environmental issues that have been linked to anthropogenic activities including fishing and tourism. Recent investigations have shown that marine litter is widely dispersed and present on Bangladeshi beaches and in nearby countries (Kaviarasan et al., 2020; Rakib et al., 2021b, 2022b). According to Rakib et al. (2022a), land-based activities, residential waste, tourists and fishing were the primary contributors of marine litter on Cox’s Bazar beach in the Bay of Bengal, Bangladesh. In a further study, Rakib et al. (2021a) found marine pollution from face masks and microplastics in the salt pans of the Moheshkhali Channel in Cox’s Bazar in a subsequent research. According to Al Nahian et al. (2023), marine litter is an anthropogenic issue that can be effectively handled once polluted hotspots are identified and proactive litter management programmes are implemented. The clean category was demonstrated by a mean Clean coast index of 4.9 for Saint Martin Island. Only 2.8% of Saint Martin’s coastline was found to be severely filthy, whereas 31% of its beaches were found to be extraordinarily clean. This baseline for Saint Martin beaches provides evidence-based data to assist with marine litter management and to encourage local community, government and non-governmental groups to begin monitoring and clean-up efforts. This fundamental study shows the magnitude of marine litter pollution, increases our understanding of coastal litter and suggests options for marine plastic management in Bangladesh’s Bay of Bengal. This study argued that the management of plastic rubbish along the shores of Saint Martin Beach in the Bay of Bengal, Bangladesh, must be tackled through educational campaigns, citizen science initiatives, behaviour modification and visitor views.

#### Nosy Be Island

Nosy Be is a tiny tourist island in northern Madagascar. As a result, the island’s resident population produces substantially more solid and liquid waste, as well as greater levels of pollution in the atmosphere and neighbouring aquatic bodies, than the island’s resident population would create on its own. This is owing to the island’s yearly visitor inflow, which is comparable to the island’s population. Tourism contributes to environmental harm on this island since there are not enough effective services and infrastructure (Mohee et al., 2015). The expanding solid waste growth on the island, in particular, is followed by a rise in the trash flow of plastics, old batteries, plastic and diapers, among other items (Chaabane et al., 2019). According to research performed by Ferronato et al. (2023) approximately 95% of Nosy Be’s trash is publicly flung, dumped to sea or openly burned. They used field investigation, interviews with local stakeholders and waste flow analysis as methodological tools to examine Nosy Be’s present SWM system. To demonstrate how touristic centres may serve as the hub of circular techniques, study findings underline the need of active participation and involvement of local partners, who are backed by foreign specialists. This case study shows how international collaboration, tourism and collaborative efforts may help low-income towns improve their SWM methods.

#### Cape Verde Island

Cape Verde is a volcanic archipelago of 10 tiny islands in the Atlantic, 570 km off the West African mainland. Cape Verde imports a huge quantity of items due to a lack of infrastructure, technology and public awareness, resulting in massive volumes of litter such as plastic that is handled (Dancette and Brêthes, 2019). As a result, waste is typically disposed of in a landfill or in inappropriate locations (e.g. the ground, valleys, etc.), raising the likelihood of being washed into the ocean. Marine litter is a major issue in Cape Verde, wreaking havoc in coastal communities, especially those that rely on marine resources for a living (Bettencourt et al., 2023). Ferreira et al. (2021) explored the attitudes of local island fishing communities in Cape Verde on marine litter in order to contribute to enhanced marine ecosystem management and the development of conservation rules. Their findings revealed the importance of public engagement and environmental education in promoting marine ecosystem conservation and the creation of robust collaborative ocean governance.

#### Mauritius Island

Mauritius is located in the South West Indian Ocean (SWIO) and has a land area of 1864 km^2^ (the main island plus neighbouring uninhabited islets), a sea area of 2.3 km^2^ and an approximate population of 1.2 million people (Seeruttun et al., 2021). Mauritius is expected to create additional waste as its population grows and its economy expands, leaving it particularly vulnerable to the consequences of marine litter. Mattan-Moorgawa et al. (2021) marine debris is a major issue that affects Mauritius’ ecosystems. They examined the density of meso-litter and microplastics in the SWIO around Mauritius Island. Plastics were revealed to be the most common type of waste. The principal sources of meso-litter were ‘shoreline and recreational activities’. The vegetation line zone has the most microplastics. The most prevalent type of microplastic was fragments that were often blue in colour, whereas polyethylene was the most common polymer. The research provides critical baseline data that may be used by competent authorities to build more effective trash management strategies and awareness campaigns to help reduce Mauritius’ marine litter problem further, as well as to analyse the efficacy of present management initiatives.

#### Fiji Islands

Fiji is composed of 332 islands, 110 of which are inhabited, and is situated in the South Pacific area of the southwest Pacific Ocean. With a few limestone or coral islands, the majority of the world’s islands are volcanic in origin. The issue of poor waste management in Fiji has gained more attention in recent years (Norrman and Soori, 2014). The absence of adequate waste management has an impact on public health, the environment, tourism and food security. The lack of comprehensive waste management is currently resulting in a number of harmful behaviours, such as illegally discarding and rubbish burning. Another significant issue of concern in Fiji is the lack of sites for the disposal of solid waste. Dumped catch, fouling incidents, net repairs, inefficiently cleaning nets and ghost fishing all have an adverse effect on fisheries. Plastics also cause extra financial losses. Marine plastics may negatively impact marine ecosystems in addition to their direct effects.

#### Samoa Islands

Samoa is made up of the two major islands of Upolu and Savai’i in addition to seven smaller islands in the Polynesian region of the South Pacific. About halfway between Hawaii and New Zealand, it is located. The island of Upolu is home to about three-quarters of Samoa’s population, as well as the nation’s capital, Apia (Gosling et al., 2020). Samoa is having serious problems handling a rising volume of more diversified waste as a result of changing lifestyles and the concentration of population in metropolitan areas. Asari et al. (2019) conducted a questionnaire study on the consumption and disposal of plastic goods at households in Samoa, one of the Pacific island countries, to better understand the flow of plastic materials and knowledge or behaviour towards plastic trash. After assessing ocean plastic contamination, they examined realistic and necessary responses in Pacific islands. They then assessed plastic ocean debris and investigated potential and necessary remedies in Pacific island countries. In Pacific island countries, the total volume of mismanaged plastic garbage was projected to be 327,000 or 156,000 tonnes. The regional Pacific island countries’ contribution to the world’s total overlooked plastic litter is projected to vary between 1.3% and 2.7%. Some Pacific island countries, such as the Solomon Islands and Micronesia, reported high levels of plastic waste mismanagement per capita. The two key reasons appear to be high rates of plastic rubbish generation and low rates of waste collection in rural areas. They emphasized that it is critical to implement measures such as improved municipal solid waste collection, reduced plastic waste, improved plastic collection and recycling and the adoption of different rules.

#### Cousine Island Seychelles

Cousine Island Seychelles is a little granitic island with an approximate height of 77 m. It is located around 2 km from Cousine Island (27 ha), a nature reserve with 20 permanent residents, and about 5 km from Praslin Island (3850 ha), the second-most populated island in the Seychelles. Small cliffs and boulders may be found on Cousine’s leeward side, but its windward, eastern side is dominated by a single, 917-m-long beach that has year-round wave activity (Samways et al., 2010). The fine-grained beach silt on Cousine is made up of fragments of calcareous exoskeletons, seashells, marine and terrestrial pebbles and broken coral. According to Dunlop et al. (2020), there is an increasing problem with ocean garbage all throughout the world, including in pristine places like Cousine Island. The nature and biodiversity of the Seychelles are significantly impacted by this expanding issue, as is tourism, which generates 60% of the nation’s income. In the Seychelles, managing solid waste is a pressing problem that need a long-term solution. Three of the most important problems in the industry include high overhead costs for waste management companies, a lack of public involvement in waste sorting and not having enough workers to handle the volume of waste produced. The problem is further made worse by the dearth of workable waste reduction techniques. Expanded polystyrene, regular glass and paper are just a few examples of additional waste categories for which waste reduction techniques, like those currently employed for polyethylene terephthalate, aluminium cans and Seychelles Brewing Company glass bottles, have been successful and can be used as a model, are available. By combining the gathering of polyethylene terephthalate, aluminium cans and glass bottles, the market for recycled goods will also be improved.

#### Cook Islands

The Cook Islands are made up of 15 islands and atolls, totalling 237 km^2^. Over 2 million km^2^, the Polynesian region of the middle Pacific Ocean has a total coastline length of 120 km. The South Pacific Ocean’s increasing plastic marine debris is bringing attention to the region’s recycling practices and restrictions (Cole and Banks, 2017). However, the economic viability of carrying it out is hampered by the special problems associated with transporting low-value commodities over long distances to recycling markets. Better waste management practices are regarded to be required for tourism to continue to be a major driver of economic growth (ADB, 2015). According to Jambeck et al. (2015), coastal towns within 50 km of the Cook Islands create 3 tonnes of plastic debris every day. On a daily basis, an estimated 1.1 tonne is incorrectly managed, infiltrating the maritime environment through direct littering or leakage from uncontained disposal sites. As a result, an estimated 416 tonnes of plastic garbage has been transformed to marine debris in Cook Islands seas in 2010. If this issue is not solved, this figure is expected to rise to 784 tonnes by 2025.

The results from the 10 discussed case studies reveal a complex landscape of marine pollution across diverse geographical locations. The Windward Islands face challenges not only from local solid waste but also from external marine and terrestrial debris. Jamaica grapples with ineffective waste disposal, with 25% of solid waste entering water bodies. Saint Martin Island showcases the need for targeted litter management programmes, emphasizing educational campaigns and behavioural modifications. Nosy Be Island in Madagascar illustrates the environmental impact of tourism on waste generation and disposal. Cape Verde faces challenges due to insufficient infrastructure, leading to extensive littering and marine pollution. Mauritius Island experiences plastic waste issues, necessitating effective management strategies and awareness campaigns. Fiji struggles with poor waste management, impacting public health, tourism and fisheries. Samoa, facing rising waste volumes, requires active participation and involvement of local partners for improved waste management. Cousine Island Seychelles highlights the adverse effects of ocean garbage on nature, biodiversity and tourism, emphasizing the need for long-term waste solutions. The Cook Islands underscore the economic and environmental challenges of recycling practices in remote regions, urging better waste management for sustained economic growth. Collectively, these case studies emphasize the urgency of implementing targeted strategies, awareness campaigns and international collaboration to address the escalating issue of marine pollution worldwide.

### Findings related to marine litter

Up to 10% of the world’s marine litter is composed of abandoned, lost or discarded fishing gear (Löhr et al., 2017). Additionally, fishing and aquaculture sectors are responsible for disposing around 0.6 million tonnes of microplastics per year (Baucher and Friot, 2017). However, the biggest contributors of marine litter are land-based activities. Particularly unregulated land-based sources cause solid waste to be carried out to sea, where it sinks to the ocean floor or floats on the surface, being carried farther away by coastal eddies and ocean currents (Consoli et al., 2018). The main land-based sources of marine litter (Galgani et al., 2015; Napper and Thompson, 2020) are (i) municipal landfills on the coast; (ii) riverine transport of solid waste from landfills or other litter sources along rivers and other inland waterways; (iii) disposal of untreated sewage from cities (especially in the sea), including storm water (produced also from occasional overflows); (iv) waste from industrial facilities, solid waste from landfills, and untreated waste water and (v) tourism and other recreational activities.

Although only around 20% of total marine litter has been classified as sea-based (Ertas 2021; Slavin et al., 2012), its importance to increase the efficiency of the current resource economy is high (European Commission, 2021), therefore justifying the need to identify the origin of marine litter. The main sea-based sources of marine litter at sea and in the ocean are: (i) commerce ships, ferries and cruise liners; (ii) fishing boats; (iii) military fleets and research vessels; (iv) pleasure watercraft; (v) offshore oil and gas platforms and (vi) fish farming (GESAMP, 2021; Veiga et al., 2016).

The top marine litter items found in European beaches include cigarette butts and filters, plastic and polystyrene pieces, food wrappers, cords, ropes and lines from fishing, caps and lids, plastic bottles, straws, cotton bud sticks and glass pieces (Addamo et al., 2017). Some items, such as fishing gear, sewage-related debris and litter left by tourists, could be confidently attributed to certain sources. Fishing nets and fishing net pieces are clear instances of items that can be directly attributed to the fishing industry, whereas cotton-bud-sticks are an example of a well-known point of origin, particularly incorrect disposal by customers (Galgani et al., 2015). However, most types of litter items are frequently impossible to be properly associated with a specific source, way of release or pathway. Some items may have multiple potential sources and ways of entry, as well as geographic origins (Veiga et al., 2016). Plastic bottles for drinks, for example, might be let on local beaches by tourists, thrown overboard by merchant shipmen, improperly disposed of on land and carried into the sea by storm water overflows. They can also reach the sea through rivers, and because they are buoyant, water currents and the dominant wind can readily carry them into a specific spot. All these factors must be taken into account for measures to reduce the amount of plastic bottles in the oceans to be successful.

The identification of the source of the items is vital. To avoid marine pollution, particular targets can be developed and actions can be taken (Pettipas et al., 2016). Preventive, mitigation and behaviour change measures to address marine litter can then be explored. According to Rangel-Buitrago et al. (2020), preventive and behaviour-changing actions address the marine litter problem at the root and are cost-effective measures with long-term impact. The preventive measures rely on reducing the generation of litter, as well as avoiding their entrance into the sea. Products’ modification (e.g. eco-design) and land-based waste management initiatives are examples of preventive actions (Chen, 2015). Jambeck et al. (2015) predicted that with no enhancements on the waste management infrastructures, the cumulative amount of land-based plastic litter entering the ocean would increase by an order of magnitude by 2025, which the most recent studies have already confirmed (Galgani et al., 2021).

However, according to Bellou et al. (2021), marine litter monitoring solutions are still undeveloped from a technology readiness perspective. In combination with slow development of clean-up solutions and mitigation strategies, results are unsatisfactory. Consequently, there is a need for development of methods, standardization and long-established and relevant ecological research (De-la-Torre et al., 2023; Haarr et al., 2022). Alongside these developments, Bettencourt et al. (2021, 2023), Corbau et al. (2023), Hartley et al. (2015) and Praet et al. (2023) argued for behaviour-changing measures, such as correct disposal of waste. These measures can be accomplished through education and raising awareness initiatives, but also require a combination of investment in waste facilities and outreach programmes (Willis et al., 2018).

Since the introduction of plastic, marine plastic litter, mainly made of SUP (Chen et al., 2021; Liu et al., 2021), has been constantly increasing (Sciutteri et al., 2023; UNEP, 2022; Wayman and Niemann, 2021). This is due to a combination of factors including population growth, irresponsible behaviour (Ronkay et al., 2021) and lack of suitable management policies. These factors together resulted in marine litter, made essentially of plastic, accumulating in rivers and oceans, spreading worldwide (Grillo and Mello, 2021) and threatening the human and ecosystem health (Raha et al., 2021; van Emmerik et al., 2022), biodiversity and marine ecosystem (Anastácio et al., 2023; Basu et al., 2021; Leal Filho et al., 2022).

Urbanization, population growth and tourism are found to be the major contributors to plastic growth, also contributing to marine litter (Basu et al., 2021; Ngoc et al., 2023; UNEP, 2022). At the same time, tourism is one of the economic activities most negatively affected by litter accumulation and incorrect waste disposal because it results in landscape degradation (Agamuthu et al., 2019; Perumal et al., 2023). Plastic production is expected to increase 40% in the next decade and so will the rate of plastic entering into the environment (Borrelle et al., 2020; Farrelly et al., 2021). These estimations already account for the numerous commitments and agreements made by both governments and industries to reduce plastic pollution (Chassignet et al., 2021). Thus, extraordinary coordinated worldwide governmental actions are needed to address the transboundary nature of plastic pollution (Farrelly et al., 2021). In Europe, although the amount of plastic waste sent to recycling had doubled, 25% of plastic post-consumer waste was still sent to landfills in 2018 (PlasticsEurope, 2021). A recent Directive (EU) 2018/852 on Packaging and Packaging Waste sets higher recycling targets per material, that is, 50% for plastic packaging by 2025 and 55% by 2030 (European Parliament and of the Council, 2018), aiming to reduce litter worldwide. This will also pose a positive impact on marine litter. However, the ever-growing marine plastic litter poses a challenge for both the collection and the subsequent plastic recyclability (Cocchi et al., 2023; Ronkay et al., 2021).

Luo et al. (2021) reported that within coastal areas, mangroves are identified as important microplastics hotspots because of the proximity to rivers and urban areas (Leal Filho et al., 2022). However, marine litter endangering these threatened vegetated coastal ecosystems are understudied, mostly focusing on area loss. Marine litter in islands tends to accumulate in larger proportions than continental sites, due to ocean transport, threatening marine wildlife (Grillo and Mello, 2021).

Hee et al. (2021) argued that while plastic in the ocean is widely researched, it mostly focuses on the fate of marine litter in the environment, being necessary to address the recycling of the materials after collection. The authors assessed marine litter’s chemical recycling through pyrolysis and gasification and energy recovery through incineration, demonstrating the available options to the treatment of the collected marine litter, offering the possibility of reintegrating plastic within the context of circular economy, so important today. In addition, Cocchi et al. (2023) showed that pyrolysis is a reliable upcycling technique to turn marine plastic litter into valuable organic compounds. Stefanini et al. (2021) argued that while contributing to the reduction of marine littering, replacing plastic with glass, for example, non-returnable glass bottles being considered the worst available option, does not contribute to the reduction of Global Warming Potential and Life Cycle Inventory, being crucial to invest in recycling and reusing, encouraging returnable packaging and raising people awareness (Mugilarasan et al., 2023; Yenici and Turkoglu, 2023), as also mentioned by Chen et al. (2021).

Studies on marine litter encompass research taking a global scope (Calvert et al., 2021; Chassignet et al., 2021; Cózar et al., 2021; Garcia-Garin et al., 2021; Povoa et al., 2021; Salgado-Hernanz et al., 2021) to specific regions (e.g. Hermawan and Astuti, 2021; Rech et al., 2021; Soto-Navarro et al., 2021; Stefanini et al., 2021; Winterstetter et al., 2021) or countries (e.g. Chowdhury et al., 2021; Loizidou et al., 2021; Mallory et al., 2021; Papakonstantinou et al., 2021; Pieper et al., 2021; Scotti et al., 2021; Yılmaz et al., 2021), addressing, among other topics, governance, policies and environmental measures and legislation, ecosystem and environmental impacts of marine plastic pollution, education and literacy, awareness and public perception or detection and quantification methods.

Education is influential to reduce plastic pollution as it leads to long-term behaviour change (Chowdhury et al., 2021; Corbau et al., 2023). Although the dangers of marine litter are recognized and well-studied, the studies show the need to go beyond, challenging the ways to address this problem in future policies to be implemented at all levels, involving governments and the communities surrounding the most affected areas. The literature shows that a holistic approach may assist policymakers and environmental experts’ collaboration in assembling a global solution to the marine litter issue, through an interdisciplinary approach that integrates consumer change for a healthy environment (Chen et al., 2021; Hoellein and Rochman, 2021; Liu et al., 2021; Luo et al., 2021; Raha et al., 2021). Still today, Europe is trying to address plastic pollution as an urgent and global problem, supporting a global agreement on plastics to end pollution by 2040 (European Commission, 2022). The negotiations on the agreement, which will be legally binding, will be finalized by 2025 (European Commission, 2022). It is expected that such an agreement will lead to the improvement of plastic pollution monitoring and regulations. It is important that the negotiations are completed on time and the agreement is widely adopted, especially in most affected areas and countries to foster the reduction of plastic pollution and marine litter.

Since China banned the import of certain wastes in 2017 (Brooks et al., 2018), developed countries have intensified their actions towards the minimization of waste generation, the same happening in some Asian countries (Malaysia, Vietnam and Thailand) in 2018. Thus, and according to Beaumont et al. (2019), actions towards marine plastic reduction within the society are absolutely necessary to defend both current and future provision of marine ecosystem services. For Agamuthu et al. (2019), addressing the issue of marine litter crisis is not a straightforward, one-size-fits-all solution, but requires a continuous effort at the local, regional and global level. Furthermore, we summarize the recommendations of areas to address in future marine litter studies. These recommendations are comprehensive, and addressing them requires concerted action, such as expressed below.

1. Data collection and monitoring systems along the supply chain (Liu et al., 2021)
2. Data collection on the coastal ecosystem types impacted by land-sourced plastic inputs (Catarino et al., 2023; Cerrillo-Escoriza et al., 2023; Harris et al., 2021, 2023)
3. iii. Standardized guidelines for macrodebris and microplastic studies (Luo et al., 2021)
4. Advance numerical models guiding decision-makers on appropriate responses (Harris et al., 2021)
5. Quantification and characterization of plastics in aquatic systems (Catarino et al., 2023; Chowdhury et al., 2021)
6. Address oceanographic characteristics and tourism infrastructure (Grillo and Mello, 2021; Ngoc et al., 2023)
7. Investment in Rs strategy and awareness (Corbau et al., 2023; Stefanini et al., 2021; Yenici and Turkoglu, 2023)
8. Identify which aspects have received the most scientific attention and to reveal overlooked pathways (Hoellein and Rochman, 2021)
9. Interdisciplinary approach and implications towards consumer driven changes (Raha et al., 2021)
10. Account measures of SUP risk and probability of exposure to that risk (Chen et al., 2021)
11. Systematic global research agenda for the recording and reporting of marine plastic research on the most vulnerable and valuable ecosystem services, and on the potential contamination of the human food chain (Beaumont et al., 2019)
12. Adoption of circular economy long-term sustainable solutions (Agamuthu et al., 2019; Cocchi et al., 2023; Mugilarasan et al., 2023; Sciutteri et al., 2023)
13. Further research on the heterogeneity and timescale of impacts, enabling the efficient development of future policies and regulations (Beaumont et al., 2019; Bettencourt et al., 2023)

### Marine litter policies, legislation and conventions

Based on the consensus that the surge of marine litter will only subside if plastics and other waste from land- and river-based sources are effectively contained, various countries adopted legal instruments to curb the plastic waste, including bans, deposits, taxes and fines on plastic products (see UNEP, 2021d for a guide on possible policy and legal approaches to curb plastic pollution; and PRI, 2023 for an interactive map of plastic legislation around the world).

Based on a study targeted at the Greek public, Charitou et al. (2021) showed that the EU Directive on SUP (2019) did not ban plastic bags, plastic bottles and other plastic packages, but required retailers to charge deposits on these products upfront and then provide refund-based recycling mechanisms. It is known that SUP production continued to increase until 2019 (Cocchi et al., 2023). Presently, and since 3 July 2021, products such as SUP plates, cutlery, straws, balloon sticks and cotton buds, cups, food and beverage containers made of expanded polystyrene and all products made of oxo-degradable plastic cannot be placed on the markets of the EU Member States (European Commission, 2021).

This plastic deposit-refund recycling policy was found successful in incentivising plastic recycling. Recycling or reusing plastic products proactively prevents them from being dumped in the environment and subsequently drifting from land and water systems into the sea (Eriksen et al., 2020; Walker and Xanthos, 2018). Guided by this strategy, the 100+ signatories of the EU Circular Plastics Alliance are committed to recycling 9 million tonnes of plastic to make new products every year in Europe by 2025 (EU, 2019). Despite EU countries’ effort in reducing plastic waste by recycling SUP, Frantzi et al. (2021) identified marine litter management in European seas as ‘post-pollution remedies’ instead of proactive policies.

The more stringent plastic bans in developing countries were found to be less successful, with some facing fierce resistance from plastic manufacturers, and the others either lacking strong or effective implementations or missing regulations on implementation (Bharadwaj et al., 2021; Clayton et al., 2021; Dhanshyam and Srivastava, 2021; Greenpeace, 2020a; Macintosh et al., 2020). China’s complete ban on plastic waste import in 2017 led to a strong decoupling of plastics consumption and economic growth in G7 and China (Wang et al., 2021), also leading to domestic management of plastic waste trade flow worldwide (Wen et al., 2021), such as banning SUP in China, to drastically reduce its massive plastic waste (Shi et al., 2021; Wang and Li, 2021; Yu and Cui, 2021). The United States, which generated the world’s largest amount of plastic waste (Law et al., 2020; Parker, 2020), belongs to the few nations that do not have federal level bans and fees on SUP, although eight states have banned SUP bags (National Conference of State Legislatures, 2021; Xanthos and Walker, 2017).

With rising awareness of the increasing marine litter and its impact (UNEP, 2022) on at least 12 United Nations (UN) Sustainable Development Goals (SDGs) (UN, 2015), a growing number of international and regional conventions, policies and legislations were adopted since the 1970s (Costa et al., 2020), such as MARPOL Convention (1973/1978), UNCLOS (1982), Basel Convention (1989), Jakarta Mandate (1995), UNFSA (1995), Stockholm Convention (2001), 2012 Manila Declaration (UNEP, 2017), 2012 Global Partnership on Plastic Pollution and Marine Litter (UNEP, 2023) and 2017 UNEP Clean Seas Campaign (UNEP, 2021a). At the regional level, conventions such as OSPAR Convention (1992), CMS (1979) and EU Directive (2000) were adopted. At the national level, countries put legislation against marine dumping in effect. In the United States, for example, the Marine Plastic Pollution Research and Control Act of 1988 prohibits dumping plastics into the ocean from any U.S. vessel or land-based operation (MARPOL Act, 1988). However, Raubenheimer and McIlgorm (2018) found the existing international and regional agreements, such as Basel Convention and Stockholm Convention, to be inadequate to manage the entire lifecycle of all plastic applications. The EU Directive (2000) was found to have issues in its implementation, such as inadequate availability, assessment and the communication between ports. No reduction was found in the litter entering the sea from land-based sources and the pollution levels (Harris et al., 2021; Pani and Pathak, 2021). This raised serious questions about the efficacy of the international and regional agreements. A new one is being prepared to help solve the problem, aiming to end plastic pollution by 2040, seeming too ambitious (European Commission, 2022).

On the other hand, the EU Marine Strategy Framework Directive (MSFD, 2008) was found to provide a sound framework to fulfil its ambitious objectives by reporting and consequently assessing (i) marine biodiversity; (ii) regional coordination and alignment of EU-relevant policies (Habitats and Birds Directives, Common Fisheries Policy, Water Framework Directive); (iii) joint monitoring programmes at the regional scale, notably for highly mobile species. However, some institutional barriers were also identified (Palialexis et al., 2021) and EU MSFD was also found to fail to monitor the deep-sea environments (Danovaro et al., 2020). EU (2019), Fadeeva and Van Berkel (2021), Steinhorst and Beyerl (2021), UNEP (2018a, 2018b) proposed to develop more effective waste avoidance and management laws, regulations, policies and action plans to encourage cities and countries, manufacturing and agricultural production and shipping, fishing and shipping fleets and the tourism industry to transform the mere ‘waste’ management to holistic ‘materials’ management in a local, national and worldwide circular economy, also contributing to drastically reduce marine litter (Cocchi et al., 2023; Sciutteri et al., 2023).

To make the current marine litter governance more effective, researchers proposed several pathways listed below (Becken et al., 2019; Costa et al., 2020; Tessnow-von Wysocki and Le Billon, 2019; US Congress, 2015; Wu, 2020; Xanthos and Walker, 2017):

1. Encompassing an inclusive treaty negotiation process
2. Respecting common but differentiated responsibilities
3. Covering plastic, microplastic, microbead wastes and chemical additives from all land- and sea-based sources
4. Promoting technological innovations for effective monitoring, reporting and review procedures
5. Developing financial incentives to support implementation measures

Several studies recommended to supplement or enhance government policies and laws through participatory engagement of multiple primary stakeholders and citizen science, phased implementation and sharing of best practices (Becken et al., 2019; Bettencourt et al., 2023; Clayton et al., 2021; McAteer et al., 2021; Tunnell et al., 2020); incentivising research on impact of marine plastic litter on seagrasses, the associated ecosystems and the food webs supported by seagrasses (Bonanno and Orlando-Bonaca, 2020); shifting marine litter policies from local-level perspectives or narrow focuses to holistic ones involving ocean and human health, and requiring dynamic strategies and adaptive management of all ocean environments (Britton et al., 2021; Khedr et al., 2023); adding massive environmental awareness campaigns, environmental training and education programmes around the world, community marine monitoring (Johnson et al., 2020), The International Coastal Cleanup (2020), zero plastic waste (CCME, 2020), EMBLAS Project (2020), Greenpeace (2020b) education programme and Regional Action Plan on Marine Litter (PAME, 2020).

Additionally, to tackle the marine litter sources from marine fishery and transportation, such as ghost nets, stronger international agreements and national legislation, exemplified by the Fishing for Litter scheme carried out in partnership with the fishing industry in Scotland and other European countries, are recommended to mandate and monitor fishermen to log the whereabouts of their fishing nets (Ronchi et al., 2019; Wyles et al., 2019), which can also result from recreational land-based fishers throwing out nets.

## Conclusions

Marine litter improperly discarded in the ocean due to poor human behaviour, unsustainable consumption and manufacturing habits, is considered to be one of the biggest global concerns that threaten ocean inhabitants and biodiversity. This problem is largely caused by human behaviour, lifestyle and lack of policy support at both national and international levels, as extensively discussed through this study. A study carried out in 16 European countries found that 95% of respondents reported encountering marine litter during their visits to beaches and coastlines. This creates urgency around the issue. Roughly 700–800 species of marine life are exposed to marine litter which may negatively affect them either through ingestion or entanglement.

Plastic is regarded as one of the most persistent and hazardous forms of marine litter. This is attributed to the lifespan, abundance and ability to break down into smaller pieces (microplastics), either equally or more hazardous than the originating item. They can release harmful toxic or hormone effective chemicals that harm animals that ingest them. Furthermore, they can absorb chemicals from the surroundings, which may be ingested by the animals.

Aside from manufacturing, the fishery industry has been implicated in the exacerbation of ocean pollution through fishing gear and other related debris. The exact contribution of fisheries to the crisis is unknown. However, recent data showed that fisheries produce 380 tonnes of waste in fishing gear per year.

The statistics surrounding marine litter are alarming. Over 100 million ocean animals die each year from plastic waste alone. The amount of plastic discarded in the ocean every year is roughly 8.3 million tonnes. Furthermore, 70% of total marine litter sinks and affects ecosystems, 15% floats around and 15% lands on beaches and coastline, and the situation is worsening.

Utilizing a comprehensive and extensive bibliometric approach, it was discovered that numerous studies and reviews have indicated unsustainable practices and a lack of supportive policies as the primary causes of marine pollution.

Findings from the 10 case studies reveal diverse challenges in marine pollution globally. Issues range from local solid waste and external debris in the Windward Islands to ineffective waste disposal in Jamaica, environmental impact of tourism in Nosy Be Island, and insufficient infrastructure leading to extensive littering in Cape Verde. The cases underscore the need for targeted solutions, awareness campaigns and international collaboration to address the escalating issue of marine pollution worldwide.

This study has limitations. The first one is the fact that the bibliometric analysis focused on publications predominantly available in English. Secondly, the focus was on aspects of plastic contamination as a whole, without a focus on specific issues such as microplastic. Despite these limitations, the study makes a valuable contribution to the literature by offering a comprehensive review of the available body of work on the topic. On this basis, the following recommendations may be made, to address the problem of ocean-based plastic pollution:

- International agreements need to be strengthened to reduce plastic waste, setting clear targets and timelines for reduction and encouraging countries to develop national action plans. In addition, legislation that bans or restricts the use of certain SUP, such as bags, straws and microbeads, need to be reinforced, so as to encourage the adoption of circular economy principles.
- Greater investment in waste management infrastructure, especially in regions near rivers and coastlines where much of the plastic enters the oceans. This includes improving waste collection, recycling and disposal facilities.
- Promotion of research and development of alternative materials that are biodegradable or more easily recycled, reducing the reliance on SUP. Here, a special emphasis may be given to bio-plastics, such as the Horizon 2020 project ‘Bioplastics Europe’ https://bioplasticseurope.eu/.
- Undertake long-term educational campaigns to raise public awareness about the impacts of plastic pollution and encourage more responsible consumption and disposal of plastics.
- Encourage companies to take responsibility for their plastic waste through extended producer responsibility schemes, where producers are responsible for the entire lifecycle of their products.

Moreover, more support and participation in clean-up initiatives is needed, to help to remove plastic from the oceans and coastlines. These can range from local beach clean-ups to larger, technology-driven efforts to remove plastic from the open ocean. Finally, increased funding and investments in both prevention and clean-up efforts are needed. This includes supporting start-ups and technologies focused on addressing plastic pollution and financing infrastructure projects in critical regions.

The UN SDGs have designed goals that specifically target marine litter and aim to result in a better aquatic life. However, governance and management regarding marine litter remain poor in many areas. Therefore, countries need to be urged to implement policies that are designed to help achieve specific goals and impose severe consequences on those that are not moving towards addressing the marine litter problem.
